# Cell death machinery makes life more robust

**DOI:** 10.7554/eLife.05816

**Published:** 2014-12-30

**Authors:** Cristina Aguirre-Chen, Christopher M Hammell

**Affiliations:** Cold Spring Harbor Laboratory, Cold Spring Harbor, United States. caguirre@cshl.edu; Cold Spring Harbor Laboratory, Cold Spring Harbor, United States, Chammell@cshl.edu

**Keywords:** development, miRNA, caspase, DIS3L2, LIN28, heterochronic, *C. elegans*

## Abstract

CED-3, a protein that is essential for programmed cell death, also has an unexpected role in the regulation of non-apoptotic genes during normal development.

**Related research article** Weaver BP, Zabinsky R, Weaver YM, Lee ES, Xue D, Han M. 2014. CED-3 caspase acts with miRNAs to regulate non-apoptotic gene expression dynamics for robust development in *C. elegans*. *eLife* **3**:e04265. doi: 10.7554/eLife.04265

**Image** Nematodes have many properties that make them ideal for investigating the gene networks that control development

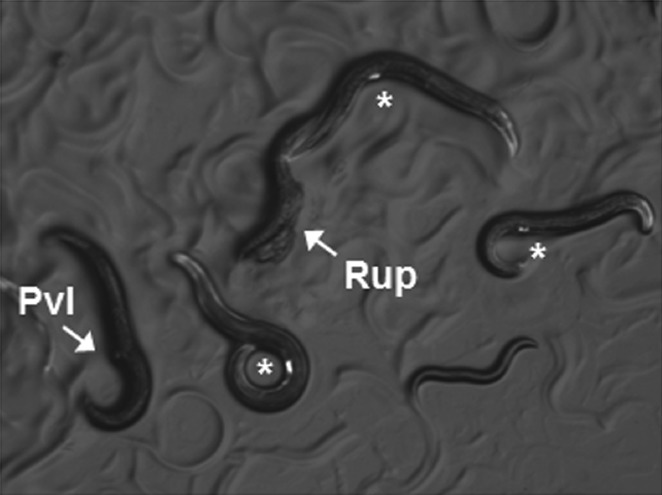

Sydney Brenner really nailed it when he proposed that the small nematode, *C. elegans*, be adopted as a model organism. The ease with which genes can be mutated in these worms, combined with the fact that all worms pass through an essentially identical sequence of events as they grow and mature, has allowed researchers to use *C. elegans* to identify genes that control development (Jorgensen and Mango, 2002). Notably, many of the subjects that *C. elegans* has become a famous model for have been studied independently, and surprisingly little overlap has been found between them. Therefore, it is extremely informative when genes central to the function of one of these processes are found to ‘moonlight’ in other gene regulatory pathways. Another example of this phenomenon has just been published in *eLife* by Min Han and co-workers at the University of Colorado in Boulder, including Benjamin Weaver and Rebecca Zabinsky as joint first authors: they have shown that a protein called CED-3, which is a key regulator of the programed cell death pathway or ‘apoptosis’, works with the machinery involved in microRNA-mediated gene regulation to control normal animal development (Weaver et al., 2014).

The first indication of this functional overlap came from a genome-wide RNAi screen, a technique used to systematically look at the effects of reducing the activity of particular genes. During normal development, the expression level of many genes involved in diverse biological processes is controlled by microRNA molecules. These microRNAs often work in large complexes. Mutations in either *ain-1* or *ain-2*, two components of the microRNA effector complex, formally known as the microRNA-Induced Silencing Complex (miRISC)*,* cause large numbers of small developmental defects (Ding et al., 2005; Zhang et al., 2007). Weaver, Zabinsky et al. have now identified 118 genes that, when their activity is reduced, make these defects much worse. These genes encode a broad spectrum of proteins, an observation that is consistent with the number of different roles that microRNAs have in regulating developmental gene expression. These ‘enhancer’ genes likely control normal gene expression in parallel with miRISC complexes (by, for example, producing transcription factors and RNA-processing components) or play important roles in maintaining the integrity of biological pathways that are regulated by microRNAs.

Conspicuous amongst this treasure trove of interesting candidates were a surprising number of genes that have been implicated in controlling apoptosis, a process by which unwanted cells are safely destroyed in a controlled, predictable way. The most striking member of this subgroup was the *ced-3* gene, which is recognized throughout biology as one of the central components of the highly conserved apoptotic pathway. The *ced-3* gene encodes a caspase—a protein that breaks down other proteins—that is found in many different species and is kept in an inactive state until needed (Miura et al., 1993; Yuan et al., 1993). Once unleashed, the CED-3 caspase is sufficient to initiate apoptosis and, until now, was thought to play a role in only this process (Xue et al., 1996; Conradt and Xue, 2005).

In a series of genetic experiments, Weaver, Zabinsky et al. combined mutant versions of the *ced-3* gene with mutations in the miRISC complex components that control microRNA-mediated gene regulation. This enhanced many of the defects that result from just compromising microRNA-mediated gene regulation. These defects are easily observable in growing animals and include those associated with the proper timing of developmental events, the formation of organs, and even behaviour. Therefore, *ced-3* and other apoptotic pathway components somehow work in a non-apoptotic role to help keep development on track.

Weaver, Zabinsky et al. then addressed the question of whether the ability of CED-3 to break down proteins is essential to its role in developmental gene regulation (Figure 1). They observed that many of the proteins whose production is regulated by microRNA molecules, including one called LIN-28, contain peptide sequences that are predicted to be CED-3 cleavage sites (Xue et al., 1996). In a series of elegant in vitro experiments, Weaver, Zabinsky et al. demonstrated three findings: that recombinant CED-3 cleaves proteins that contain these sites; that cleavage is inhibited by caspase-specific inhibitors; and that mutating the CED-3 cleavage site found in LIN-28 prevents cleavage from occurring. Next they asked whether expressing a version of LIN-28 that CED-3 cannot break down would reproduce many of the developmental and genetic changes seen when *ced-3* activity is lost. They found that expressing the modified version of LIN-28 not only caused the nematodes to grow more slowly, it also altered the order or patterns in which specific developmental processes normally occur.

**Figure 1.**
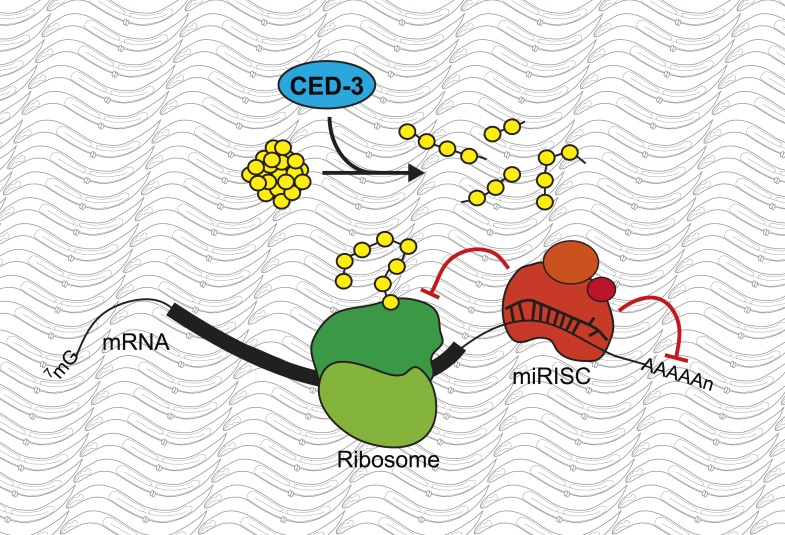
A new role for the caspase CED-3. Modifications to messenger RNA (mRNA) molecules can influence the extent to which a given gene is expressed. For example, microRNA molecules, which are found in miRISC complexes (red), can bind to the mRNA molecule and stop them from being translated into proteins by molecular machines called ribosomes (green), or induce their degradation. Weaver, Zabinsky et al. propose a model whereby these regulatory activities of the microRNAs in a miRISC complex are enhanced by the CED-3 caspase (blue) breaking down proteins (yellow) after they have been translated. This acts as a further level of regulation, and so makes it more likely that developmental gene expression is robustly controlled and the organism will develop correctly.

As with all provocative hypotheses, the proposal put forward by Weaver, Zabinsky et al. raises a number of additional questions. For example, how conserved is this mechanism in biology? Are the protein products of major microRNA targets enriched for putative CED-3 cleavage sites? How many of the remaining 117 candidates derived from the *ain-1 or ain-2* enhancer screens are going to be as interesting or as exciting as *ced-3*? Given the breadth and complexity of microRNA-mediated gene regulation, we can be sure that there will be several.
